# Regional differences in the associations of diet quality, obesity, and possible sarcopenia using the seventh Korea National Health and Nutrition Examination Survey (2016-2018)

**DOI:** 10.4178/epih.e2023059

**Published:** 2023-06-19

**Authors:** Hyeongyeong Lee, Sohyun Park

**Affiliations:** 1 Department of Food Science and Nutrition, Hallym University, Chuncheon, Korea; 2 The Korean Institute of Nutrition, Hallym University, Chuncheon, Korea

**Keywords:** Sarcopenia, Obesity, Diet, Healthy Eating Index, Hand grip strength

## Abstract

**OBJECTIVES:**

Sarcopenic obesity is closely related to aging and the prevalence of various chronic diseases and frailty. The purpose of this study was to analyze whether diet quality is related to obesity, sarcopenia, and sarcopenic obesity, and if so, to explore the difference in that relationship between urban and rural settings.

**METHODS:**

Using data from the Korea National Health and Nutrition Examination Survey of 2016-2018, a total of 7,151 participants aged 40 years or older were analyzed. Sarcopenia was diagnosed using handgrip strength. Diet quality was assessed using Korea Healthy Eating Index (KHEI) scores, and obesity was determined based on participants’ abdominal circumference. Multinomial logistic analysis was used for testing statistical significance.

**RESULTS:**

Rural participants had significantly lower KHEI scores and a higher prevalence of sarcopenic obesity than urban participants. The study findings demonstrate that participants without obesity, sarcopenia, or sarcopenic obesity had significantly higher KHEI scores in both rural and urban settings. Multinomial regression analysis further revealed that a higher KHEI score was associated with a lower risk of sarcopenia and sarcopenic obesity among urban residents, while only the risk of obesity was lower with higher diet quality scores among rural residents.

**CONCLUSIONS:**

Since diet quality and health status were lower in rural areas, it is important to address this regional disparity with appropriate policy measures. To mitigate urban health disparities, urban residents in poor health with few resources should also be supported.

## GRAPHICAL ABSTRACT

[Fig f2-epih-45-e2023059]

## INTRODUCTION

Body composition changes with age, including loss of muscle and an increase in body fat [1,2]. Muscle loss can lead to major health issues, such as a higher risk of falls in the elderly [3]. A study using US National Health and Nutrition Examination Survey data showed that among the elderly, the prevalence of functional disorders was higher in participants with a low muscle index (muscle mass divided by body weight, as a percentage) [4].

Sarcopenia is a musculoskeletal disease that has received increasing medical attention and was classified as a disease in 2016 by the International Classification of Diseases, 10th revision, Clinical Modification [5]. Sarcopenia is significantly associated with physical dysfunction [6], falls [7], and the prevalence of metabolic disorders [8]. Sarcopenia has also been shown to predict mortality [9].

Sarcopenia is generally diagnosed using 3 criteria that reflect skeletal muscle function: muscle strength, muscle mass, and physical performance. Muscle strength is considered the most important characteristic of sarcopenia by the European Working Group on Sarcopenia in Older People (EWGSOP) [10]. Low muscle strength has been termed probable sarcopenia by the EWGSOP [10] and possible sarcopenia (PS) by the Asian Working Group for Sarcopenia (AWGS) [11]. It is underscored that effective intervention at early stages can prevent, delay, and treat sarcopenia [10,12]; early diagnosis can also reduce medical costs. According to United States data from a study published in 2004, the direct medical costs of sarcopenia exceeded 18 billion dollars per year [13].

In numerous cases, muscle mass decreases and body fat increases with aging. In a study that monitored the effects of aging on body composition for 10 years, body fat increased in both males and females as age increased [14]. Changes in body composition, such as increased body mass index (BMI), body fat, and abdominal visceral fat, are associated with not only a risk of functional and physical disorders but also cardiovascular disease and type 2 diabetes [15,16]. Sarcopenic obesity includes both sarcopenia, in which muscle mass and muscle strength decrease, and increased body fat. Studies have shown associations between sarcopenic obesity and lower physical function [17], mild cognitive impairment, and dementia [18]. Hence, sarcopenic obesity is a crucial health issue due to its close association with negative health outcomes in the elderly.

Diet plays a critical role in the risk and prevention of sarcopenia. Vitamin D and protein have protective effects against the muscular loss and functional impairment associated with aging, including sarcopenia [19,20]. Although it has been reported that the probability of frailty is lowest with a higher Mediterranean diet score [21,22], an association has not yet been established between sarcopenia and dietary patterns or quality. A previous study emphasized the importance of sufficient protein intake in every meal [23], and diverse dietary patterns improved handgrip strength (HGS) and gait speed [24]. It may be important to improve the overall quality of meals, rather than increase consumption of certain nutrients.

Previous studies have reported regional variations in diet quality. Specifically, the diets of elderly patients residing in rural areas are comparatively worse than those in urban areas, even after adjustment for socio-demographic variables [25]. Furthermore, regional differences are seen for various health indices, and in numerous cases, disease prevalence and health behavior are worse in rural areas [26-28]. Despite such variance in diet quality or health indices, regional studies examining associations between diet quality and sarcopenic obesity are still lacking. Therefore, our study aimed to examine the association of obesity and PS with diet quality in adults aged 40 years or older. The effect of regional differences on this association was also examined.

## MATERIALS AND METHODS

### Data and participants

**Figure 1.**
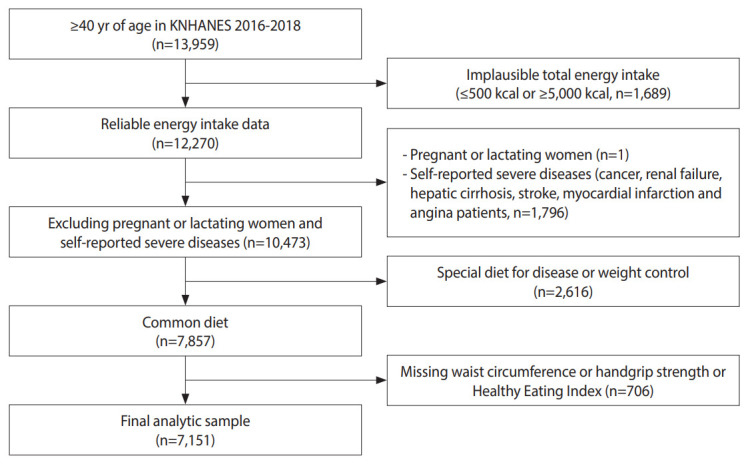
Study participants flow chart, Korea National Health and Nutrition Examination Survey (KNHANES) 2016-2018.

The Korea National Health and Nutrition Examination Survey (KNHANES) is a program resulting in nationally representative data about health and food consumption behavior in Korea [29]. This study analyzed data from the KNHANES. Given that obesity and sarcopenia tend to develop after age 40, our study focused on participants aged 40 and older [30]. Individuals were excluded from the analysis if their energy intake was ≤ 500 kcal or ≥ 5,000 kcal (n=1,689), if they were pregnant or lactating (n=1), or if they had diseases that may affect diet, such as cancer (n=883), renal failure (n=53), hepatic cirrhosis (n=47), stroke (n=386), and myocardial infarction and angina (n=427). Additionally, individuals on a special diet for disease or weight control (n=2,616) were excluded, as were those whose records lacked waist circumference (n=398), HGS (n=307), or Korean Healthy Eating Index (KHEI) information (n=1). Data from 7,151 participants were used for analysis (Figure 1).

### Measurement of muscle strength and assessment of sarcopenia

Among various methods of assessing muscle strength, large-scale studies have commonly used HGS measurements [31]. The EWGSOP guidelines recommend diagnosing PS using HGS [10]; The KNHANES also measured HGS to assess the muscle strength of participants. The HGS indices included in data from the seventh KNHANES were measured with a digital grip strength dynamometer (TKK 5401 Grip-D; Takei, Japan) in participants aged 10 years or older. HGS was measured 6 times (3 times per hand), and the maximum value among the 6 measurements was used in this study. According to the AWGS 2019 criteria, men whose maximum HGS was 28 kg or lower and women whose maximum HGS was 18 kg or lower were classified as having PS.

### Assessment of diet quality

Diet quality was assessed using KHEI scores. The KHEI consists of 14 items based on domestic guidelines for healthy eating habits: 8 items evaluating the adequacy of recommended food and nutrient intake (KHEI adequacy score), 3 items evaluating the intake of restricted food and nutrients (KHEI moderation score), and 3 items evaluating the balance of energy intake (KHEI energy balance score). The total score is calculated by adding the scores from each item, and a higher KHEI indicates an overall higher diet quality [32,33]. This allows the evaluation of overall eating habits and diet quality in Korean adults.

### Assessment of obesity

Obesity is commonly diagnosed by BMI or abdominal circumference. Since middle-aged and elderly adults were the subjects of our analysis, obesity was determined based on abdominal circumference, which better reflects body fat distribution in both groups and predicts morbidity and mortality more effectively [34,35]. Abdominal circumference ≥ 85 cm and ≥ 90 cm was used to define obesity for women and men, respectively [36].

### Statistical analysis

The variables used in the analysis are area (urban or rural); household type; household income; education level; health behaviors such as smoking, alcohol consumption, and physical activity; food security; and the number of chronic diseases. For area, households in *dong* (neighborhood) administrative divisions were classified as urban, and households in *eup* (town) and *myeon* (township) divisions were classified as rural.

Household type was classified as “single member” or “non-single.” Household income was classified into quartiles (lowest, lower middle, upper middle, and highest). Education level was classified into 4 groups (elementary school or less, middle school, high school, and college and above). Smoking status was classified as “current smoker,” “past smoker,” and “never.” Alcohol consumption was classified as “no” for those who reported drinking less than 1 glass per month over the past year or never having consumed alcohol. Physical activity was categorized as “yes” for those who performed moderate physical activities weekly for at least 2 hours and 30 minutes, high-intensity physical activities weekly for at least 1 hour and 15 minutes, or a mixture of moderate and high-intensity physical activities (1 minute at high intensity equals 2 minutes at moderate intensity) for the corresponding durations per week. Food security was classified as “sufficient,” “quality insufficient” when participants responded that they did not have diverse food items, and “quality and quantity insufficient” when participants responded that they lacked sufficient foods due to financial hardship. Chronic disease was classified into 0-1, 2, and 3 by the number of diseases with which the participants were diagnosed, among diabetes, hypertension, and dyslipidemia.

When analyzing the data, sampling weights were used to reflect the complex sample design specified in the data usage guide of the seventh KNHANES [37]. Categorical variables were described in frequency and percentage, and the chi-square test was applied. Linear regression analysis was used to compare the KHEI mean scores according to type of residential area and the presence of obesity and sarcopenia. The means and standard errors (SEs) of KHEI scores were presented after adjusting for age and gender. For the analysis of odds ratios (OR) of sarcopenia and obesity according to KHEI scores, multinomial logistic regression was used with adjustment for age, gender, household type, household income, education level, health behaviors (smoking, drinking, physical activities), food security status, and the presence of chronic disease, which were all found to be associated with obesity and sarcopenia status and type of residential area. All analyses were conducted using Stata/MP 17.0 (StataCorp., College Station, TX, USA) with 2-tailed 95% confidence intervals (CIs) and a statistical significance threshold of p< 0.05.

### Ethics statement

The study protocol was approved by the Institutional Review Board of Hallym University (HIRB-2021-087) and Korea Centers for Disease Control and Prevention (KCDC) (waived from 2015 to 2017, and 2018-01-03-P-A).

## RESULTS

The general characteristics of the participants by type of residential area are shown in Table 1. Among the 7,151 participants, 22.7% resided in rural areas. The percentage of residents aged 65 years or older was higher in rural (44.7%) than in urban areas (30.2%). The proportion of single-person households was higher in rural areas, and education level was higher among urban residents. There was no difference in the percentage of current smokers between urban and rural areas, but alcohol consumption and participation in physical activity were higher in urban than rural residents. There was no difference in food security status. In rural areas, more participants had 2 or more chronic diseases compared to urban residents. Obesity, sarcopenia, and sarcopenic obesity were more prevalent in rural than in urban areas. For KHEI scores reflecting diet quality, the total scores (65.37 vs. 63.39), adequacy score (32.39 vs. 30.91), and energy balance score (8.97 vs. 7.88) were significantly lower in rural than in urban residents.

The general characteristics of the participants according to obesity and sarcopenia are shown in Table 2. In the sarcopenia and sarcopenic obesity groups, the percentages of participants aged 65 years or older were higher than for participants aged 40-64 years old. The proportions of sarcopenia and sarcopenic obesity were higher in the groups with lower household income, and they were highest in the group with the lowest education level. A comparison of health behaviors showed that the smoking rate was the highest in participants with obesity, and the drinking rate was lower in participants with sarcopenia and sarcopenic obesity compared to participants with obesity. Physical activity also gradually decreased from the normal group to the sarcopenic obese group. For food security, the response rate of “quality and quantity insufficient” was highest in the sarcopenic obesity group. Having 2 or more chronic diseases was most likely in the group with sarcopenic obesity.

A comparison of KHEI scores according to the presence of sarcopenia and obesity in both types of areas is shown in Table 3. In urban residents, the mean KHEI total score was 66.33±0.28 in the normal group, 65.22±0.37 in the obesity group, 61.22±0.81 in the sarcopenia group, and 60.18±0.92 in the sarcopenic obesity group. In rural residents, the KHEI total score was 64.26±0.60 in the normal group, 62.56±0.75 in the obesity group, 60.69±1.12 in the sarcopenia group, and 59.34±1.65 in the sarcopenic obesity group. The KHEI total score was lowest in the sarcopenic obesity groups in both urban and rural areas. In both urban and rural areas, the highest KHEI adequacy scores were found in normalweight participants, followed in descending order by participants with obesity, sarcopenia, and sarcopenic obesity. The highest KHEI energy balance score was found in the normal group, followed in descending order by the obesity, sarcopenia, and sarcopenic obesity groups, but with statistical significance in urban areas only.

The results from the multinomial logistic regression models are presented in Table 4. ORs are shown for obesity, sarcopenia, and sarcopenic obesity compared to the normal group, with respect to KHEI scores in both urban and rural areas. Among urban residents, an increase of 1 unit in KHEI scores was associated with a significant decrease in the odds for sarcopenia (OR, 0.98; 95% CI, 0.97 to 0.99) and sarcopenic obesity (OR, 0.97; 95% CI, 0.95 to 0.98), while the OR for obesity did not show statistical significance. Conversely, among rural residents, an increase of 1 unit in KHEI scores was associated with a significant decrease in the odds for obesity (OR, 0.98; 95% CI, 0.97 to 0.99), but not for sarcopenia or sarcopenic obesity. The interaction terms between diet quality and obesity/sarcopenia status were not statistically significant, indicating that the association between diet quality and obesity/sarcopenia did not differ by type of residential area.

## DISCUSSION

Diet plays a crucial role in the risk and prevention of sarcopenia; however, studies analyzing the correlation between dietary patterns and diet quality are lacking [38]. Moreover, previous studies have shown that there is a significant disparity in diet quality and the risk of disease in different areas, even within a country [26]. Therefore, this study aimed to examine whether the association of KHEI scores with sarcopenia and obesity differed depending on participants’ residential area using KNHANES data. In both urban and rural areas, diet quality was the highest in the normal group and the lowest in the sarcopenic obese group (Table 3). This is consistent with previous studies reporting that the prevalence of sarcopenia was lower in men elderly patients who had higher index-international scores of diet quality [24].

Our results showed that rural residents showed a higher prevalence of obesity, sarcopenia, and sarcopenic obesity than urban residents. Since this reflects the crude prevalence without age adjustment, this finding could have been due to a higher proportion of elderly people in rural areas. Key health behaviors and health indicators also showed some negative values among rural participants, along with other variables of socioeconomic status. This aligns with previous studies showing disparities in frailty and aging-related health indicators between urban and rural areas [28]. Discussions of health disparities between urban and rural areas are ongoing outside of Korea as well [27].

Diet quality, measured with the KHEI, was also significantly lower in rural participants. In detail, the largest difference was in KHEI adequacy scores, and urban areas also scored higher than rural areas for KHEI energy balance. Other studies have shown that individual gender, age, education level, behaviors such as smoking, and obesity rate were associated with KHEI scores [39]. However, this relationship may also result from differences in the local food environment, as well as various socio-demographic and economic variables. In particular, the food environments experienced by the elderly in urban and rural areas are bound to be different. Even with the adjustment of variables that affect individual eating habits, diet quality is poorer in the elderly residents of rural areas than in elderly urban residents. Our study is in line with previous studies analyzing factors affecting diet quality in Korea’s urban and rural areas [25].

It is widely acknowledged that the food environment, especially the accessibility of food, is sparser in rural than in urban areas, as shown by both domestic and international studies [39,40]. Access to grocery stores can be difficult in rural areas with a low population density, and ingredients are less fresh, reducing customers’ satisfaction [41]. Considering the lack of public transportation and lower income status in many rural areas, it can be quite challenging to access food there [42]. This implies that community-level interventions are necessary to improve the rural food environment and thus the diet quality among rural residents. These factors may affect future health indicators such as obesity and sarcopenia.

Our study showed that those with both obesity and sarcopenia had the worst health behaviors and the lowest socio-demographic status compared to participants with either obesity or sarcopenia alone. Similarly, a previous study reported that the presence of sarcopenic obesity had more negative effects on elderly individuals than the presence of only obesity or sarcopenia [17]. It has been reported that larger quantities of body fat exerted direct and indirect effects on the balance of muscle amino acids by inducing the expression of tumor necrosis factor, which can alter protein metabolism via alterations to insulin sensitivity [43]. Another study has shown that muscle loss induces insulin resistance by reducing the mass of insulin response target tissues, which promotes metabolic syndrome and obesity [44]. An increase in fat and a loss of muscle may act together to trigger disorders and morbidity synergistically [45]. It was reported that compared to sarcopenia, sarcopenic obesity is associated with lower physical function [17], and the risk of cardiovascular disease increased in the sarcopenic obesity group by 23% compared to the normal group, whereas the risk did not significantly increase in the sarcopenia or obese groups [46]. In addition, a study has shown that obesity in older individuals was associated with a decline in muscle mass and quality of life [47]. These results suggest that sarcopenic obesity, as a combination of sarcopenia and obesity, may pose an even greater risk for aging individuals.

In both rural and urban areas, the KHEI scores for overall diet quality were highest in the normal group, followed in descending order by the normal, obesity, sarcopenia, and sarcopenic obesity groups. To understand whether this pattern of a linear decrease in diet quality is the same in urban and rural areas, the significance of the interaction term was analyzed. The result was statistically significant (Table 3), which suggests that health indicators such as sarcopenic obesity have a higher correlation with KHEI scores for urban residents. Although few studies have examined health disparities within urban areas, their results have suggested that inequities in medical care caused by income differences in urban areas may be relevant [48]. A study using KNHANES data showed that only some differences in chronic disease management between urban and rural areas were statistically significant; however, when urban residents were compared to one another, more statistically significant differences were found in chronic disease management. Hence, larger gaps in income and education level among urban residents may lead to larger discrepancies in health indicators, compared to rural residents, who have been found to be more homogeneous in terms of socioeconomic indices [49].

A comparison of the normal group and sarcopenic obesity group showed large differences in age (56.0 vs 71.7 years) as well as other socioeconomic indices (Table 2). Increased age can lead to a transition from a normal weight to obesity, sarcopenia, or sarcopenic obesity. Since our analysis was a cross-sectional study, it is hard to determine which factor was the most relevant for the increasing prevalence of sarcopenic obesity as individuals grew older. Therefore, longitudinal studies are needed to understand which factors other than aging contribute most to sarcopenic obesity occurring through the aging process.

To our knowledge, this is the first study examining the association of overall diet quality with probable sarcopenia and sarcopenic obesity, measured with simple HGS. Our study confirmed that it is feasible for sarcopenia-related studies to follow the advice by the AWGS that PS can be assessed by HGS, which allows the evaluation of muscle function without a complicated X-ray examination. Diet quality was found to be consistently lower among participants from rural areas compared to their urban counterparts, which may be used as basic evidence to establish policies that can enhance diet quality among rural residents. However, the correlation of diet quality with sarcopenic obesity and sarcopenia was more prominent among urban residents than among their rural counterparts. Since there is abundant evidence suggesting that sarcopenic obesity may worsen with aging and may have negative effects on physical function, interventions for vulnerable elderly individuals in urban areas also need to be developed.

Despite the strengths of this study, a few limitations should be considered when interpreting the results. Our study included participants aged 40 years or older because obesity and sarcopenia begin to develop at this age [30]. Since our main research goal was to examine KHEI scores and their association with sarcopenia and obesity, stratified by type of residential area, we did not further stratify the participants based on age. Although all the statistical models were adjusted for gender and age, it would be meaningful to perform additional analyses to determine whether the patterns are similar when the models are stratified by participants’ ages. In addition, a causal relationship could not be determined, reflecting an inherent limitation of cross-sectional studies.

Our study results provide insights into the associations of diet quality with obesity, sarcopenia, and sarcopenic obesity among representative Korean middle-aged and elderly adults in both urban and rural areas. Future studies are warranted to investigate dietary interventions that may reduce sarcopenia, obesity, and sarcopenic obesity, with tailored strategies for rural and urban residents.

## Figures and Tables

**Figure f2-epih-45-e2023059:**
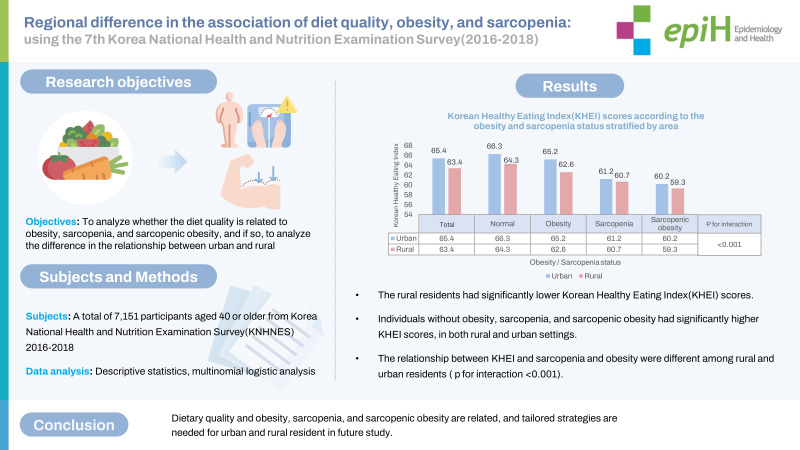


**Table 1.** General characteristics of study participants by residential area

Characteristics		Total (n)	Urban	Rural	p-value^1^
Age (yr)					<0.001
	40-64	4,757	3,859 (77.6) [0.75]	898 (65.8) [2.36]	
	≥65	2,394	1,667 (22.4) [0.75]	727 (34.2) [2.36]	
Gender					0.204
	Men	3,133	2,402 (51.0) [0.69]	731 (53.2) [1.60]	
	Women	4,018	3,124 (49.0) [0.69]	894 (46.8) [1.60]	
Household type					<0.001
	Single member	941	654 (8.8) [0.53]	287 (13.8) [1.32]	
	Non-single	6,210	4,872 (91.2) [0.53]	1,338 (86.2) [1.32]	
Household income					<0.001
	Lowest	1,643	1,075 (15.8) [0.77]	568 (30.9) [2.56]	
	Lower middle	1,733	1,283 (21.8) [0.80]	450 (26.4) [1.58]	
	Upper middle	1,749	1,428 (27.8) [0.83]	321 (23.2) [1.96]	
	Highest	2,005	1,723 (34.5) [1.21]	282 (19.5) [1.87]	
Education level					<0.001
	≤Elementary school	1,913	1,245 (18.4) [0.78]	668 (34.9) [2.31]	
	Middle school	870	633 (10.9) [0.54]	237 (15.2) [1.39]	
	High school	2,047	1,666 (33.5) [0.88]	381 (31.1) [1.90]	
	≥ College	1,964	1,734 (37.3) [1.23]	230 (18.9) [1.76]	
Smoking					0.016
	Current smoker	1,219	931 (20.7) [0.74]	288 (25.1) [1.58]	
	Past smoker	1,618	1,266 (25.1) [0.66]	352 (22.6) [1.30]	
	Never	4,241	3,279 (54.2) [0.73]	962 (52.3) [1.88]	
Alcohol consumption					0.001
	No	3,492	2,601 (43.2) [0.80]	891 (49.8) [1.77]	
	Yes	3,590	2,875 (56.8) [0.80]	715 (50.2) [1.77]	
Physical activity					<0.001
	No	4,259	3,148 (58.5) [0.93]	1,111 (70.0) [1.64]	
	Yes	2,523	2,122 (41.5) [0.93]	401 (30.0) [1.64]	
Food security					0.991
	Sufficient	3,773	2,920 (54.1) [1.09]	853 (53.8) [2.10]	
	Quality insufficient	3,160	2,435 (43.1) [1.06]	725 (43.4) [2.04]	
	Quality and quantity insufficient	218	171 (2.8) [0.32]	47 (2.8) [0.75]	
No. of chronic diseases^2^					0.060
	0-1	6,046	4,714 (87.7) [0.49]	1,332 (85.0) [1.21]	
	2	892	659 (10.1) [0.46]	233 (12.0) [1.12]	
	3	213	153 (2.2) [0.23]	60 (2.9) [0.43]	
Sarcopenic obesity status					<0.001
	Normal	4,293	3,452 (64.7) [0.83]	841 (54.7) [1.43]	
	Obesity	1,801	1,362 (24.9) [0.76]	439 (28.1) [1.38]	
	Sarcopenia	613	418 (6.1) [0.37]	195 (10.3) [0.93]	
	Sarcopenic obesity	444	294 (4.2) [0.31]	150 (6.8) [0.76]	
Korea Healthy Eating Index score, mean±standard error					
	Total	65.03±0.21	65.37±0.23	63.39±0.49	<0.001^3^
	Adequacy	32.15±0.18	32.39±0.19	30.91±0.43	0.002^3^
	Moderation	24.09±0.08	23.99±0.09	24.59±0.19	0.005^3^
	Energy balance	8.79±0.07	8.97±0.08	7.88±0.18	<0.001^3^

Values are presented as unweighted number (weighted %) [standard error].

1 Using the chi-square test, accounting for the complex survey design.

2 The number of chronic disease was categorized into 3 groups according to the number of diseases diagnosed among hypertension, diabetes, and dyslipidemia.

3 Using regression models after taking into account the complex survey design.

**Table 2.** General characteristics of participants according to their obesity and sarcopenia status

Characteristics		Total (n)	Normal	Obesity	Sarcopenia	Sarcopenic obesity	p-value^1^
Area							<0.001
	Urban	5,526	3,452 (85.6) [1.44]	1,362 (81.6) [1.89]	418 (74.9) [2.92]	294 (75.4) [3.04]	
	Rural	1,625	841 (14.4) [1.44]	439 (18.4) [1.89]	195 (25.1) [2.92]	150 (24.6) [3.04]	
Age (yr)							<0.001
	40-64	4,757	3,318 (83.8) [0.64]	1,169 (74.9) [1.28]	182 (36.2) [2.40]	88 (25.1) [2.59]	
	≥65	2,394	975 (16.2) [0.64]	632 (25.1) [1.28]	431 (63.8) [2.40]	356 (74.9) [2.59]	
Gender							<0.001
	Men	3,133	1,902 (51.9) [0.85]	894 (58.1) [1.36]	172 (30.0) [2.33]	165 (39.3) [2.82]	
	Women	4,018	2,391 (48.1) [0.85]	907 (41.9) [1.36]	441 (70.0) [2.33]	279 (60.7) [2.82]	
Household type							<0.001
	Single member	941	444 (8.0) [0.53]	239 (9.5) [0.82]	153 (19.1) [1.55]	105 (17.9) [2.00]	
	Non-single	6,210	3,849 (92.0) [0.53]	1,562 (90.5) [0.82]	460 (80.9) [1.55]	339 (82.1) [2.00]	
Household income							<0.001
	Lowest	1,643	659 (12.6) [0.69]	433 (19.7) [1.21]	316 (46.9) [2.57]	235 (48.4) [3.09]	
	Lower middle	1,733	1,022 (22.2) [0.86]	462 (22.9) [1.28]	137 (23.4) [2.06]	112 (25.7) [2.49]	
	Upper middle	1,749	1,162 (28.7) [0.92]	448 (28.3) [1.46]	85 (15.5) [1.78]	54 (14.2) [2.23]	
	Highest	2,005	1,440 (36.6) [1.24]	454 (29.1) [1.49]	71 (14.1) [1.92]	40 (11.7) [2.10]	
Education level							<0.001
	≤Elementary school	1,913	766 (14.3) [0.67]	533 (22.5) [1.21]	335 (52.3) [2.76]	279 (62.9) [2.90]	
	Middle school	870	519 (10.9) [0.55]	241 (12.8) [0.97]	61 (11.5) [1.59]	49 (13.5) [2.18]	
	High school	2,047	1,393 (35.9) [0.95]	500 (32.1) [1.50]	99 (22.6) [2.19]	55 (14.3) [1.98]	
	≥College	1,964	1,426 (38.8) [1.21]	453 (32.7) [1.63]	57 (13.6) [2.12]	28 (9.3) [1.89]	
Smoking							<0.001
	Current smoker	1,219	761 (22.1) [0.82]	350 (23.8) [1.36]	64 (12.4) [1.63]	44 (11.7) [2.08]	
	Past smoker	1,618	954 (24.4) [0.76]	471 (28.0) [1.36]	103 (18.4) [1.93]	90 (19.5) [2.14]	
	Never	4,241	2,548 (53.5) [0.87]	966 (48.2) [1.46]	428 (69.2) [2.37]	299 (68.8) [2.84]	
Alcohol consumption							<0.001
	No	3,492	1,931 (41.3) [0.94]	839 (40.9) [1.36]	426 (70.9) [2.23]	296 (65.3) [2.74]	
	Yes	3,590	2,332 (58.7) [0.94]	948 (59.1) [1.36]	172 (29.1) [2.23]	138 (34.7) [2.74]	
Physical activity							<0.001
	No	4,259	2,380 (56.2) [1.03]	1,151 (64.8) [1.40]	407 (72.4) [2.36]	321 (77.6) [2.47]	
	Yes	2,523	1,720 (43.8) [1.03]	576 (35.2) [1.40]	140 (27.6) [2.36]	87 (22.4) [2.47]	
Food security							<0.001
	Sufficient	3,773	2,345 (55.4) [1.12]	968 (54.5) [1.56]	255 (43.6) [2.47]	205 (48.5) [2.82]	
	Quality insufficient	3,160	1,843 (42.4) [1.10]	782 (42.4) [1.53]	326 (50.9) [2.51]	209 (45.5) [2.90]	
	Quality and quantity insufficient	218	105 (2.1) [0.31]	51 (3.1) [0.60]	32 (5.4) [1.09]	30 (6.0) [1.25]	
No. of chronic diseases^2^							<0.001
	0-1	6,046	3,860 (91.4) [0.49]	1,383 (81.1) [0.99]	518 (85.7) [1.54]	285 (66.1) [2.81]	
	2	892	368 (7.5) [0.46]	319 (14.3) [0.88]	78 (11.8) [1.46]	127 (27.3) [2.63]	
	3	213	65 (1.1) [0.16]	99 (4.6) [0.55]	17 (2.6) [0.65]	32 (6.5) [1.61]	

Values are presented as unweighted number (weighted %) [standard error]. Normal, non-sarcopenia and non-obesity; Obesity, non-sarcopenia and obesity; Sarcopenia, sarcopenia and non-obesity; Sarcopenic obesity, sarcopenia and obesity.

1 Using the chi-square test, accounting for the complex survey design.

2 The number of chronic diseases was categorized into 3 groups according to the number of diseases diagnosed among hypertension, diabetes, and dyslipidemia.

**Table 3.** Korean Healthy Eating Index (KHEI) scores according to obesity and sarcopenia status and stratified by residential area^1^

KHEI total scores		Urban					Rural					p for interaction
		Normal	OB	SAR	SAR OB	p for trend	Normal	OB	SAR	SAR OB	p for trend	
Total		66.33±0.28	65.22±0.37	61.22±0.81	60.18±0.92	<0.001	64.26±0.60	62.56±0.75	60.69±1.12	59.34±1.65	0.002	<0.001
	Adequacy	33.08±0.23	32.57±0.32	28.86±0.61	28.17±0.66	<0.001	31.64±0.50	30.58±0.78	28.79±0.99	26.98±1.23	<0.001	
	Moderation	24.16±0.10	23.77±0.18	24.22±0.27	24.21±0.34	0.589	24.29±0.23	23.70±0.30	24.26±0.47	24.50±0.54	0.508	
	Energy balance	9.09±0.10	8.88±0.14	8.14±0.28	7.81±0.34	<0.001	8.32±0.20	8.27±0.27	7.65±0.41	7.86±0.45	0.171	

Values are presented as mean±standard error. Normal, non-sarcopenia and non-obesity; OB, non-sarcopenia and obesity; SAR, sarcopenia and non-obesity; SAR OB, sarcopenia and obesity.

1 Means and p for trend are obtained using generalized linear regression models after adjusting for age and gender.

**Table 4.** Korea Healthy Eating Index (KHEI) scores and odds ratios for obesity, sarcopenia, and sarcopenic obesity, stratified by residential area^1^

Obesity/Sarcopenia status	Urban	Rural	p for interaction
Normal	1.00 (reference)	1.00 (reference)	
Obesity	0.99 (0.99, 1.00)	0.98 (0.97, 0.99)	0.147
Sarcopenia	0.98 (0.97, 0.99)	0.99 (0.97, 1.01)	0.375
Sarcopenic obesity	0.97 (0.95, 0.98)	0.99 (0.97, 1.01)	0.286

Values are presented as odds ratio (95% confidence interval). Normal, non-sarcopenia and non-obesity; Obesity, non-sarcopenia and obesity; Sarcopenia, sarcopenia and non-obesity; Sarcopenic obesity, sarcopenia and obesity.

1 The explanatory variable was KHEI in a continuous format; Odds ratios were obtained using multinomial logistic regression models after adjusting for age, gender, household type, household income, education, smoking, drinking, physical activity, food security status, and the number of chronic diseases; The odds ratios can be interpreted as the risk for obesity, sarcopenia, or sarcopenic obesity relative to normal status if a subject’s KHEI score were to increase by 1 unit, with the other variables in the model held constant.
